# Trust-based partner switching among partitioned regions promotes cooperation in public goods game

**DOI:** 10.1371/journal.pone.0253527

**Published:** 2021-06-28

**Authors:** Hongwei Kang, Mie Wang, Yong Shen, Xingping Sun, Qingyi Chen

**Affiliations:** School of Software, Yunnan University, Kunming, China; University of Electronic Science and Technology of China, CHINA

## Abstract

In this paper, the coevolution mechanism of trust-based partner switching among partitioned regions on an adaptive network is studied. We investigate a low-information approach to building trust and cooperation in public goods games. Unlike reputation, trust scores are only given to players by those with whom they have a relationship in the game, depending on the game they play together. A player’s trust score for a certain neighbor is given and known by that player only. Players can adjust their connections to neighbors with low trust scores by switching their partners to other players. When switching partners, players divide other nodes in the network into three regions: immediate neighbors as the known region, indirectly connected second-order neighbors as the intermediate region, and other nodes as the unknown region. Such choices and compartmentalization often occur in global and regional economies. Our results show that preference for switching to partners in the intermediate region is not conducive to spreading cooperation, while random selection has the disadvantage of protecting the cooperator. However, selecting new partners in the remaining two regions based on the average trust score of the known region performs well in both protecting partners and finding potential cooperators. Meanwhile, by analyzing the parameters, we find that the influence of vigilance increasing against unsatisfactory behavior on evolution direction depends on the level of cooperation reward.

## 1 Introduction

Cooperative behavior is ubiquitous in biological systems and economic societies [1]. The functioning of society is inseparable from the cooperation and connection between individuals. However, explaining the cooperative behavior of unrelated individuals in human society and animal communities is still an open question [2,3]. Based on the prisoner’s dilemma game (PDG) model [4] and its various modified versions [5,6], this problem has been extensively studied. In this game, two players decide simultaneously on either cooperation (C) or defection (D). They both receive an amount R upon mutual cooperation and P upon mutual defection. A defector exploiting a cooperator gets an amount T and the exploited cooperator receives S. The usual setting is T>R>P>S, which means that when acting in one’s own interests, it is best to defect regardless of the opposing player’s strategy. Consequently, for any scenario that imitates Darwinian selection, cooperation should not exist [7]. The public goods game (PGG) [8–11] can be interpreted as an n-person prisoner’s dilemma game. In this game, a cooperator sacrifices part of his own resources to contribute to the public goods, but a defector does not contribute to others. Although cooperative behavior can maximize the payoff of the group, rational individuals still choose defective behavior to improve their own interests. Therefore, for rational individuals, free riding is the dominant strategy, which leads to collective irrationality, referred to as the tragedy of the commons [12]. However, cooperation persists in real societies. Therefore, various mechanisms that can promote cooperation have been studied, including punishment [13–15], exclusion [16,17], kin selection [18], group selection [19–21], direct reciprocity [22–25], indirect reciprocity [26–28], and network reciprocity [29,30].

Nowak and May introduced the regular lattice network as a framework that allowed players to play PDGs only with their own immediate neighbors [29]. In this work, they found that spatial structure can promote cooperation to some extent. However, Hauert et al. found that the regular lattice network does not always promote cooperation, especially in the snowdrift game [31]. Inspired by studies on spatial games, researchers started to pay attention to how topologies influence cooperation results. Many complex networks have been studied extensively, such as random networks [32,33], small-world networks [34–36], and scale-free networks [37–43], which are closer to the structured populations in reality. The above-mentioned research on network topology is mostly carried out on static networks, but the relationships between individuals are not constant. Therefore, there has also been a surge in research on the coevolution game with dynamic social networks [44–48]. Players are allowed to update strategies, end old relationships or establish new connections in order to find a mutually beneficial partnership.

The coevolutionary game can be driven by aspiration [49,50] or empathy [51], reputation [26,52]. Reputation has been shown to have a strong influence on cooperative results in dynamic games [26], which models indirect reciprocity in human societies. In addition to theoretical models, empirical studies also reveal the positive role played by reputation. Sylwester and Roberts conducted economic experiments, and the experimental results showed that investing in cooperative reputation and selecting partners based on reputation could bring net benefits [53]. Notably, this definition of reputation in most studies is similar to image scoring proposed by Nowak and Sigmund, which could be regarded as an affine transformation of an image [26]. Reputation is a non-revenue system of reward for players who choose to cooperate during evolution, which is used by other players to determine whether a player is worth cooperating with. In some studies, player reputation is conditionally acquired by other players. Fu et al. proposed the mechanism of reputation-based partner switching, in which players are allowed switch from the lowest reputation partners to their partners’ highest reputation partners [54]. In this work, the player knows the reputation value of his partner and his partner’s partners. However, after switching, the historical value of reputation will still be held by the new partner(s). Reputation, similar to a social credit policy, becomes a player’s social attribute. Interestingly, in some cases, accurate social reputation information about new partners is not available. Therefore, we discuss the establishment of cooperation and trust using a simpler model. At the same time, since partner selection and evaluation often exist among major economic regions, we also analyze the influence of new partner selection on the evolution of cooperation based on trust scores.

In this paper, we propose the mechanism of trust-based partner switching among partitioned regions. In our model, trust is established between players who have a game relationship. Trust is established by the mutual evaluation of two players in the form of a trust score. It needs to be emphasized that players can only evaluate their partners based on future games they will play together. A player who gives a high trust score to a neighbor indicates satisfaction with the payoff generated by the neighbor. Mutually satisfied players’ relationship will be stable. Meanwhile, the player disconnects from neighbors based on a low trust score, which is a topological punishment for unsatisfactory neighbors. For players to find more suitable new neighbors when switching partners, players divide the network into three areas based on how much information they have. Take player *x* as an example. Immediate neighbors have a trust score since they were directly connected to player *x*; this is the known region. Second-order neighbors that are not directly connected are the intermediate region, who are connected to the player *x*’s immediate neighbors. Other nodes in the network can be considered the unknown region by player *x*. When reselecting a neighbor from the three regions, the player will make full use of the limited trust score information in the intermediate region, which reflects the average level of cooperation in the player’s own environment.

The rest of the paper is structured as follows. Section 2 introduces the coevolution model of cooperation. Section 3 specifically analyzes the results through numerical simulation of the model. Finally, in Section 4, we discuss some implications of our findings and future work.

## 2 Model

**Fig 1:**
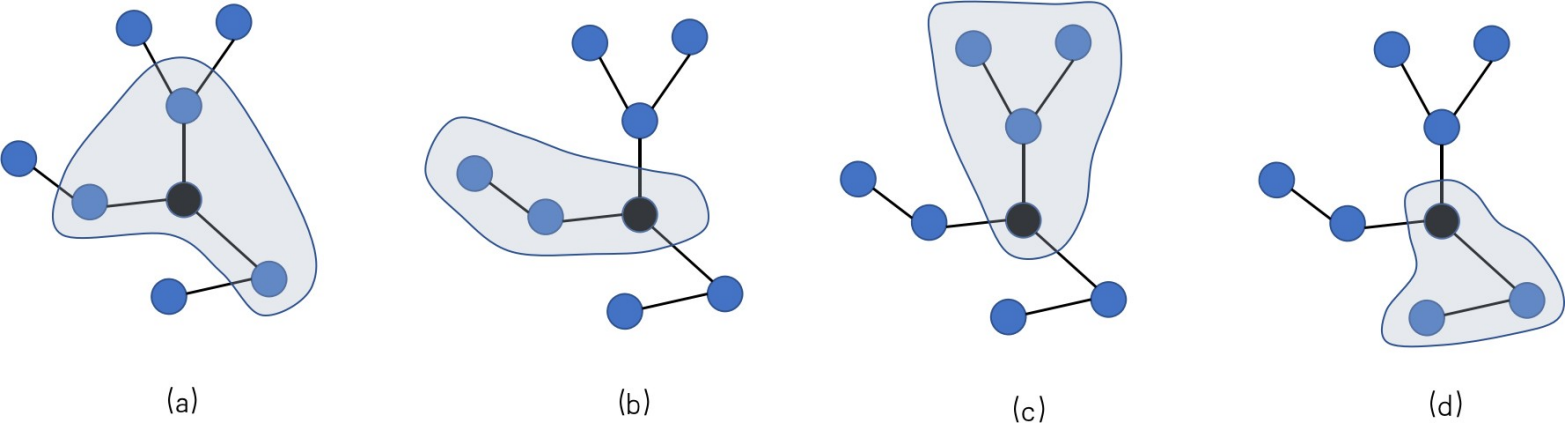
An example of public goods game on the network. We show 8 structured populations, where each node is connected to other nodes. Each player participates in a public goods game centered on him and his neighbors. For example, the black nodes in the graph participated in four public goods games: one centered on itself (a), and the other three centered on its three neighbors (b)-(d). The payoff of the central node comes from four public goods games. Payoff for the other players is calculated in the same way.

Let us first define the population structure and dynamic rules. On a dynamic graph consisting of nodes and edges, games are played in the neighborhood centered on each node(player). Each node *i* of degree *k*_*i*_ participates in *k*_*i*_+1 public goods games, played between itself and its *k*_*i*_ immediate neighbors, as shown in Fig 1. A player’s total payoff is accumulated from each game played. Before introducing the dynamic rule driven by trust scores, let us look at the player’s unilateral static payoff analysis. Assuming *x* as the center player, for one of the neighbors *y*, what *x* obtains from *y* can be expressed as:

$$e_{xy}=\frac{rs_{y}}{k_{x}+1},s_{y}=\left\{ \begin{array}{c} 1\;y\;is\;C\\ 0\;y\;is\;D \end{array}\right.$$ (1)

where *r* denotes the synergy factor of investment. In each round of the game, cooperators contribute an investment c to the public goods, and the benefits of the total investment will be shared equally among all players in the group. Obviously, if neighbor *y* chooses to cooperate, *x* will be one of the beneficiaries in *k*_*x*_+1 and take the benefit *e*_*xy*_ which is part of the value-added benefit of *y*’s cost c in common pool. In this paper, the total investment in each game for each cooperator is set to 1. To calculate the unilateral payoff, in addition to the benefit *x* gets from his neighbor y, the cost of *x*’s investment on the *x-y* edge has to be calculated. Similarly, the value given to *y* by *x* can be expressed as:

$$c_{xy}=\frac{s_{x}}{k_{x}+1},s_{x}=\left\{ \begin{array}{c} 1\;x\;is\;C\\ 0\;x\;is\;D \end{array}\right.$$ (2)

Eq 2 indicates that the cost of player x’s investment in the common pool will be equally invested in each neighbor. Therefore, in each game centered on *x*, for the *x-y* edge, the unilateral payoff of *x* can be expressed as:

$$P_{xy}=e_{xy}-c_{xy}$$ (3)

**Fig 2:**
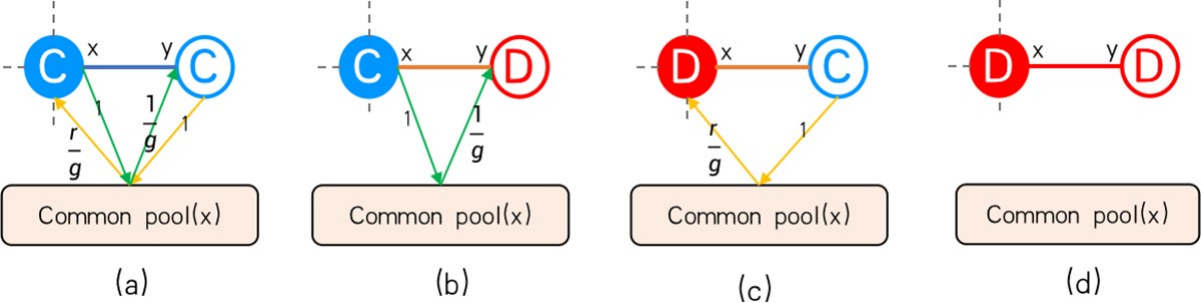
Four cases of the center node *x* and the neighbor node *y*. In four cases, the amount that the central node *x* receives from neighbor *y* and the cost to neighbor *y* are analyzed. The green arrow shows the amount that the center node gives to the neighbor node, and the yellow arrow shows what the center node gets from the neighbor node. A red node indicates that the node is a defector, and blue indicates that the node is a cooperator. *g* is the number of players in the game centered on *x*.

In each game, both the centered player and its neighbors have two alternative strategies: cooperation or defection. For different combinations of strategies, we summarize four possibilities, as shown in Fig 2. Meanwhile, we analyzed the unilateral payoff [55] of the center player *x* in Table 1 for four combinations of an *x*−*y* edge. The total payoff of player *x* can be expressed as the sum of all neighbors’ unilateral payoff:

$$P_{x}=\sum_{y\in \Omega_{x}^{\prime }}P_{xy}$$ (4)

where $\Omega_{x}^{\prime }$ is the set of *x*’s neighborhood, including all neighbors of *x*.

**Table 1:** Unilateral static payoff analysis of a central player in a separate game.

*x-y*	*e*_*xy*_	*c*_*xy*_	*P*_*xy*_
*C-C*	$\frac{r}{k_{x}+1}$	$\frac{1}{k_{x}+1}$	$\frac{r}{k_{x}+1}-\frac{1}{k_{x}+1}$
*C-D*	0	$\frac{1}{k_{x}+1}$	$-\frac{1}{k_{x}+1}$
*D-C*	$\frac{r}{k_{x}+1}$	0	$\frac{r}{k_{x}+1}$
*D-D*	0	0	0

Where *x* is the central player, *y* is a neighbor of *x* and *k*_*x*_ is the degree of the central player. The strategy chosen by *x* and *y* is denoted by C (Cooperation) or D (Defection). In the table, when *x* and *y* choose different strategies in the game, the unilateral payoff of *x* in edge *x-y* will be shown. Note that this is after one round of game.

In order to demonstrate the partner switching mechanism, we define the trust score *T*_*xy*_ of a player *x* for its immediate neighbor, which can be fully understood in mathematical terms. The value of unilateral payoff is a fair reference factor for updating the trust score. Therefore, each player’s trust score for its neighbors is unbiased. A player’s trust score for its neighbors is initialized at 0, including for new neighbors gained in the coevolution process. For ease of calculation, we fix the range of the trust score, $\hat{T}\in [-T,T],(T>0)$. After each game centered on *x*, *x* updates each neighbor *y’s* trust score as:

$$T_{xy}(t)=\left\{ \begin{array}{l} T_{xy}(t-1)+\alpha ∙H(P_{xy})-\beta ∙H(-P_{xy}),\;P_{xy}\neq 0\\ T_{xy}(t-1)-\varepsilon ∙\beta ,\;P_{xy}=0 \end{array}\right.$$ (5)

0≤ε<β

where *H(x)* is the Heaviside function, which distinguishes three unilateral payoff cases. We consider three trust scores, -*β* for a neighbor whose unilateral-payoff is negative in round *t*; *α* for a neighbor whose unilateral-payoff is positive in round *t*; and −*β∙ε* for neighbor who unilateral payoff is 0 in round *t*. We use *ε* to represent the decline rate of the trust score when centered player gets nothing from the neighbor. We set *ε* = 0.2 in the paper. Simply put, the player’s trust in each neighbor depends on the payoff brought by the neighbor, not on the neighbor’s strategy. The player updates each neighbor’s trust score in round *t*, then starts partner switching and strategy updating. *α*, *β*, and *β∙ε* respectively represent different attitudes towards the three kinds of benefits brought by neighbors. The three parameters are simple abstractions of the evaluation of the unilateral benefits of the neighbor. This is also very common in real life, where we give positive comments and strengthen relationships with partners who consistently bring in positive revenue; Instead, our perception of the partner who is making a loss continues to decline and we may choose to replace the unsatisfied partner. In our model, strategy and topology are updated synchronously: the time scale of the strategy update is equal to that of the topology update. Players use the latest trust score to determine whether each neighbor is worthy of continuing their partnership connection. After the structure updates, the player is allowed to choose a neighbor to learn its strategy with a certain probability. Partner switching and strategy updating are the two main dynamic rules in the coevolution model in this paper.

The player’s satisfaction for a partnership can be expressed by the trust score given to a neighbor. By identifying trust scores of neighbors, the player can switch partners dynamically, which creates an evolving adaptive network. Neighbors with lower trust scores are more likely to be switched, but partners with higher scores are more likely to be retained. We use the cumulative Poisson distribution to describe the probability *F* of switching a partner:

$$F=\sum_{m=0}^{\left\lfloor {-T}_{xy}(t)\rfloor H({-T}_{xy}(t)\right)}\frac{e^{-\lambda }\lambda^{m}}{m!}$$ (6)

The parameter *m* refers to the partner *y’s* trust score. *λ* is the expectation of the probability distribution, which states that the probability of switching the neighbor will be close to 0.5 when *m* = *λ*. In this paper, we set *λ* = 10. This means that neighbors with a trust score greater than 0 have a near zero probability of being removed, and the probability of edge breaking increases when the score begins to be less than 0. In other words, the probability of a neighbor being switched increased as its trust score decreases. In this model, connectivity requirements are not taken into account when edges are broken, as isolation during evolution is also a part of natural selection.

**Fig 3:**
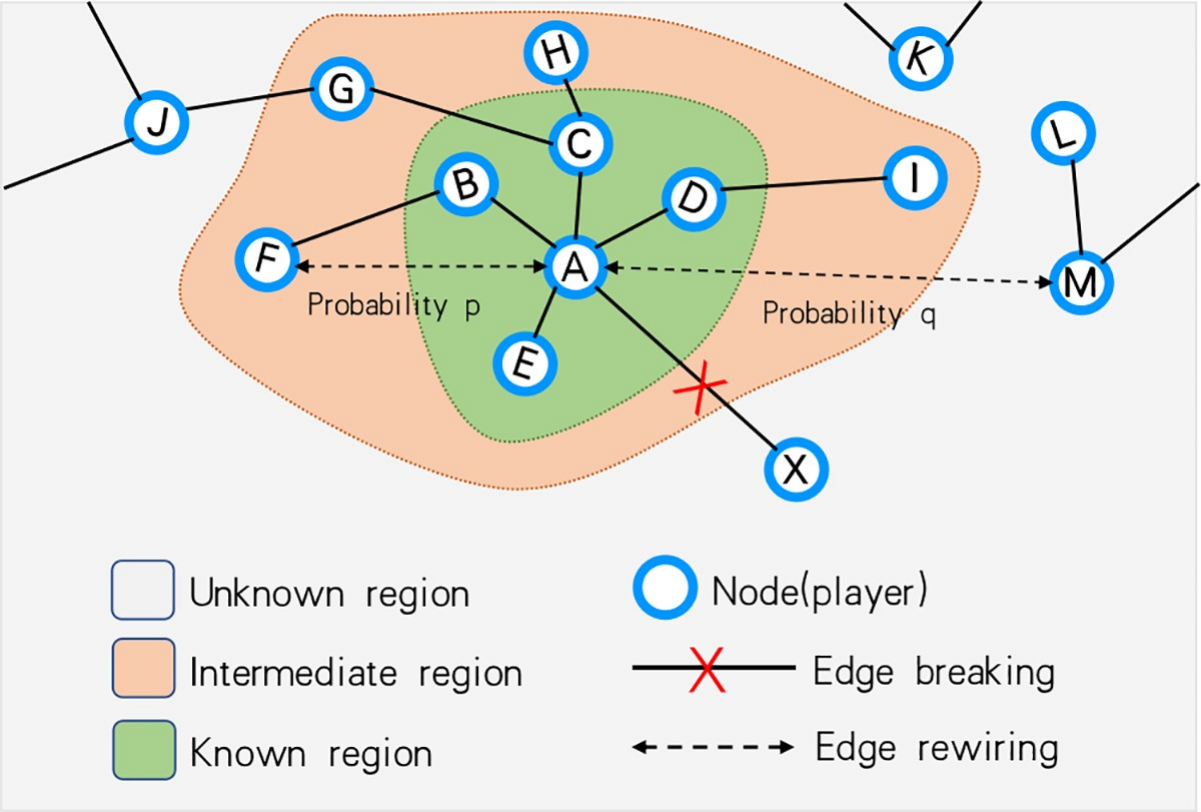
Example of rewiring of partner switching. Node *A* disconnects the edge of node *X*. Node *A* is allowed to divide other nodes into three regions. The immediate neighbors are the known region (nodes *A-D*), shown in green. Node *A* has played with players in the known region and has given them a trust score. The second-order neighbors that are not directly connected (*F-I*), shown in orange, make up the intermediate region. Other nodes (*J-M*) belong to the unknown region and are shown in gray. The probability of the node selecting a new partner in intermediate region is *p*, and the probability of selecting a partner in the unknown region is *q* = 1-*p*. *p* is a function of the average trust score of the known region.

Players who choose to disconnect a neighbor will be allowed to select a new node as a new partner. Each player has a trust score for each neighbor, which partly reflects the level of cooperation in the environment in which the player is located. Using this information, the whole network can be divided into three areas by player as shown in Fig 3: the direct neighborhood is the known region; second-order neighbors who are not directly connected is the intermediate region; the remaining region is unknown. If the average trust score in the known region is higher, the player is more inclined to choose a new partner in the intermediate region. However, once the average trust score of the known region is less than 0, players will not consider choosing a new partner in the intermediate region. The probability of a player *x* with degree *k* choosing a new partner in the intermediate region is *p*:

$$p=Tanh\left(\frac{1}{k}∙\sum_{y\in \Omega_{x}}T_{xy}\right)∙H\left(N_{s}∙\sum_{y\in \Omega_{x}}T_{xy}\right)$$ (7)

*Ω*_*x*_ is the set of immediate neighbors of player *x*. *Tanh(x)* is a monotonically increasing activation function with a range of (0,1), widely used in Machine-learning. The parameter *N*_*s*_ in the Heaviside function is the number of second-order neighbors of player *x*. This means that the player can only choose a new partner in the unknown region when the known region trust score is less than 0 or there is no node in the intermediate region.

Players update their strategies by imitating their linked partners. Take for example *x*, who selects a random partner *y* and imitates their strategy with probability:

$$W_{s_{x}\to s_{y}}=\frac{1}{1+exp(-\frac{P_{x}-P_{y}}{\omega })}$$ (8)

where *ω* is the extent of noise, which reflects some irrational factors. Precisely, *ω*→∞ leads to random drift while *ω*→0 is absolute rationality. In this paper, we set *ω = 0*.*1*.

## 3 Simulation result

In this section, we show in detail the impact of trust-based partner switching among partitioned regions on public goods games. We start from the initialization of the lattice network with *N* players, where all players have the same number of edges. It should be emphasized that although there are few cases of lattice networks in real social networks, the simple initial network more intuitively reflects the influence of the trust score-driven coevolution on cooperation. In our partner switching model, players will be allowed to select a new partner after removing a neighbor, which keeps the network average degree unchanged. Meanwhile, players have no strategic preferences when choosing new partners, so we do not consider isolated defectors in this paper. Initially, 50% cooperators and 50% defectors are randomly distributed in the network. The level of cooperation in the simulation is reflected by the fraction of cooperators in all the players. The data in Figure is the average of 30 independent runs. The number of cooperators in each run is obtained from the results of 10,000 Monte Carlo simulations.

### 3.1 Impact of the network

**Fig 4:**
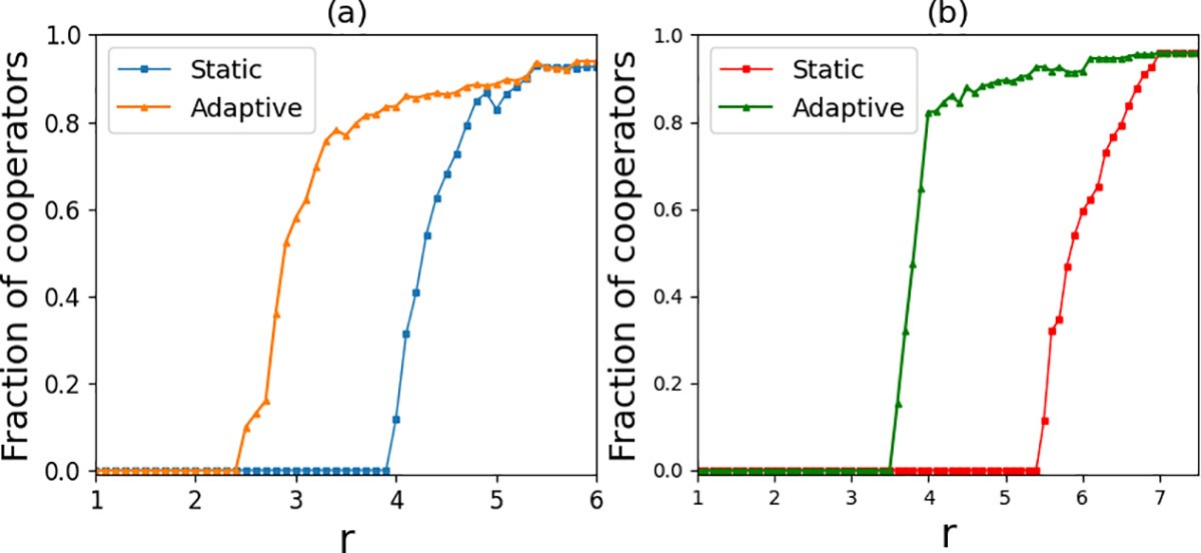
Fraction of cooperators as a function of the synergy factor r for coevolution adaptive network and static regular network. (a) Average degree of network $\hat{k}=4$; (b) average degree $\hat{k}=6$. Under two average degrees, cooperative behavior emerges earlier with the partner switching mechanism than in a static network. Parameters: *N* = 10000, *t* = 10000, *T = 20*, *α* = 5, *β* = 5, and *ω* = 0.1.

In this paper, each player (node) in the initialized network has the same number of game partners (edges), which means the whole simulation begins with a regular network with an average degree of $\hat{k}$. In static networks, Ohtsuki et al. concluded that $b/c>\hat{k}$ is the condition under which natural selection is conducive to cooperative evolution in degree rule networks [30]. Here *c* is the cost of cooperation, *b* is the benefit of cooperation, and $\hat{k}$ is the degree of the network. In order to illustrate the influence of network average degree $\hat{k}$ on the partner switching coevolution model, we compared the results of cooperation under different $\hat{k}$. As shown in Fig 4, in the static network without promotion mechanism, the emergence of cooperation at $\hat{k}=4$ is earlier than $\hat{k}=6$. However, the cooperation of the two regular networks both resulted in the synergy factor $r=\hat{k}$ approximately, which further verified the conclusions of Ohtsuki et al [30]. In addition, in the coevolutionary network in this paper, cooperation also emerges earlier when $\hat{k}=4$ compared with $\hat{k}=6$. This means that a high average degree will increase the defector’s advantage to some extent. It should be noted that, regardless of whether $\hat{k}=4$ or $\hat{k}=6$, the emergence of cooperation in the partner switching coevolutionary network is much earlier than that in the static network with the same average degree. Therefore, partner switching among partitioned regions can increase the advantage of the cooperator. In other words, the cooperation strategy can survive with a smaller *r* in our model than in a static network with the same average degree.

**Fig 5:**
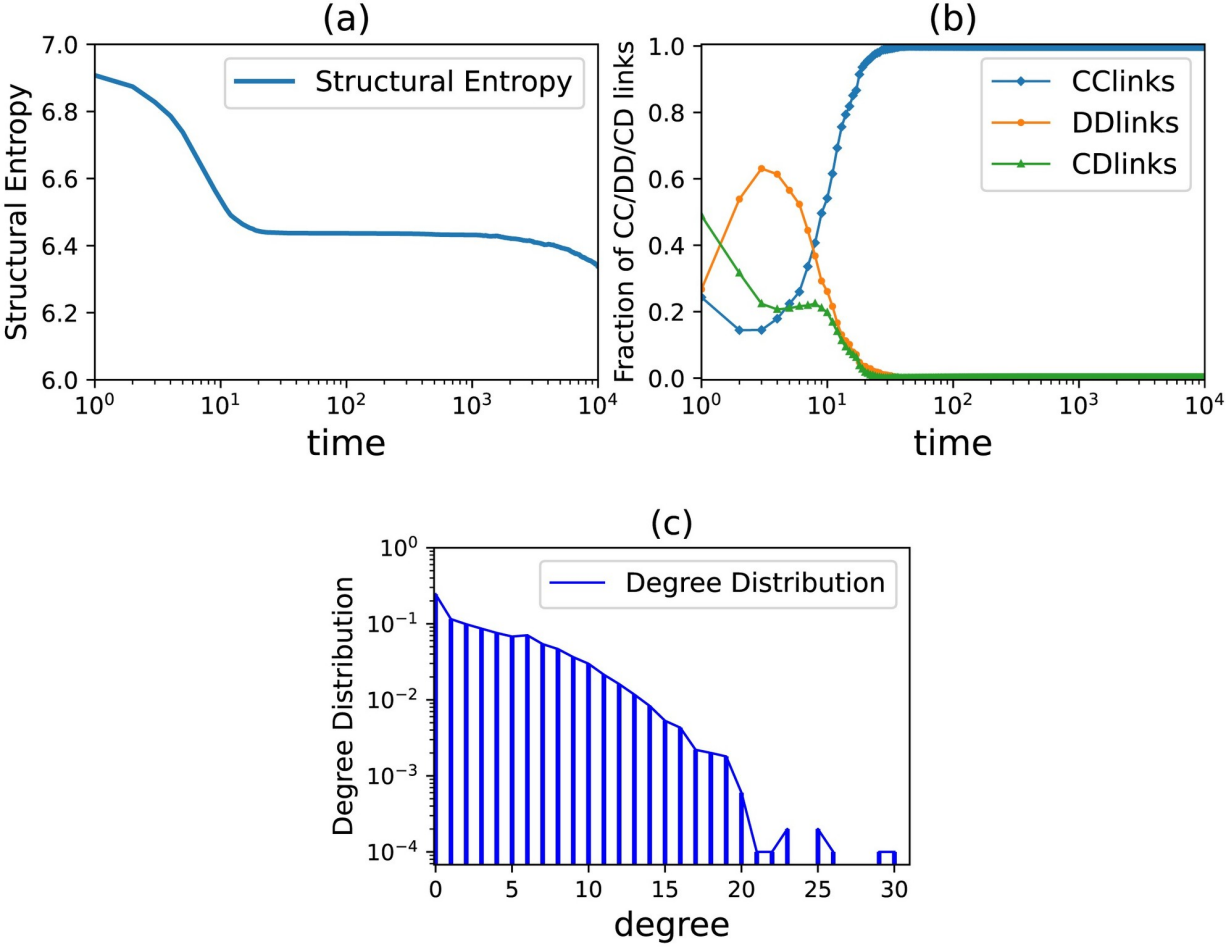
The changes of network indicators during the evolution of partner switching. (a) Time evolution of network structure entropy, (b) The time evolution of CC/CD/DD connection fraction in network, (c) Degree distribution in the evolutionarily stable network. *N* = 10000, *t* = 10000, *T* = 20, *α* = 5, *β* = 5, $\hat{k}=4$ and *ω* = 0.1.

In order to further analyze the changes and influences of the network in the evolution process, we continue to explore the changes of various indicators of the network under the condition that $\hat{k}=4$. As shown in Fig 5(B), in the coevolution model under the rules in this paper, the edges of *DD* and *CD* in the network are significantly inhibited and eventually evolve into full cooperation. Meanwhile, we also introduce the network structure entropy to analyze the network topology information in the evolution process. Entropy is a physical concept that can reflect the chaos within the system. The structural entropy of a network is defined by the relative number of nodes with the same degree value in the network. To some extent, the network structure entropy can reflect the network topology information. The structural entropy of the network can be expressed as:

$$E=-\sum_{i}p(i)\;\log\;p(i)$$ (9)

where *p*(*i*) is the probability distribution of the node degree of the network, reflecting the relative number of nodes with the same degree in the network. Node degree probability distribution *p*(*i*) can be expressed as:

$$p(i)=\frac{k_{i}}{\sum_{j}\;k_{j}}$$ (10)

As shown in Fig 5(A), we find that during the evolution process of partner switching, the network structure entropy continues to decline, but the rate of decline gradually slows down. In the early stage of evolution, the network structure is very unstable, which leads to great changes in the network structure. However, in the later stage of evolution, the network structure tends to be stable in the adaptive network with partner switching, and the decreasing rate of network entropy also decreases. After the evolution stabilizes, as shown in Fig 5(C), compared with the initial regular network, the node degree of the network begins to show obvious differentiation, indicating that the partner switching mechanism will increase the network heterogeneity.

### 3.2 The role of trust score

As described in Section 2, when a player switches neighbors, it will select a new partner from either the intermediate region or the unknown region according to the average trust score of the known region. In a simple simulation of the average degree analysis, we find that this mechanism allows cooperation to survive in a smaller r, as shown in Fig 4. In order to examine the effect of coevolutionary trust-based partner switching among partitioned regions, we explore the cooperation results under different regional preference choices. Preference choices refer to which region the player prefers to choose new neighbors from when switching partners. We conduct a comparative study on the cooperative results of three preferred partner switching modes. In addition to the trust-based partner switching model described in Section 2, we propose two partner switching options: intermediate region preference and random choice. It should be emphasized that differences exist only in the edge rewiring method among the three models, while the decision method for edge-breaking is consistent. In the three models, we use *p* to represent the probability of choosing a new partner in the intermediate region.

*Model-I*, *trust-based*. This model has been described in detail in Eq (6). When players rewire, *p* is determined by the average trust score of players in known region (directly connected players). This model makes full use of the trust score information of the known region.

*Model-Ⅱ*, *intermediate region preference*. Players in this model will preferentially choose one node in the intermediate region as new partner when rewiring. Unless the number of second-order neighbors *N*_*s*_ is 0, the new partner is randomly selected in the unknown region. The probability *p* in *Model-Ⅱ* is expressed as follows:

$$p=H(N_{s})$$ (11)

*Model-Ⅲ*, *random choice*. In this model, the player will first consider the nodes in the whole network after breaking an edge. Similarly, the probability of choosing a new partner in the intermediate region is only related to the number of nodes *N*_*s*_. The probability *p* with random choice can be expressed as:

$$p=\frac{N_{s}}{N-k_{x}}$$ (12)

**Fig 6:**
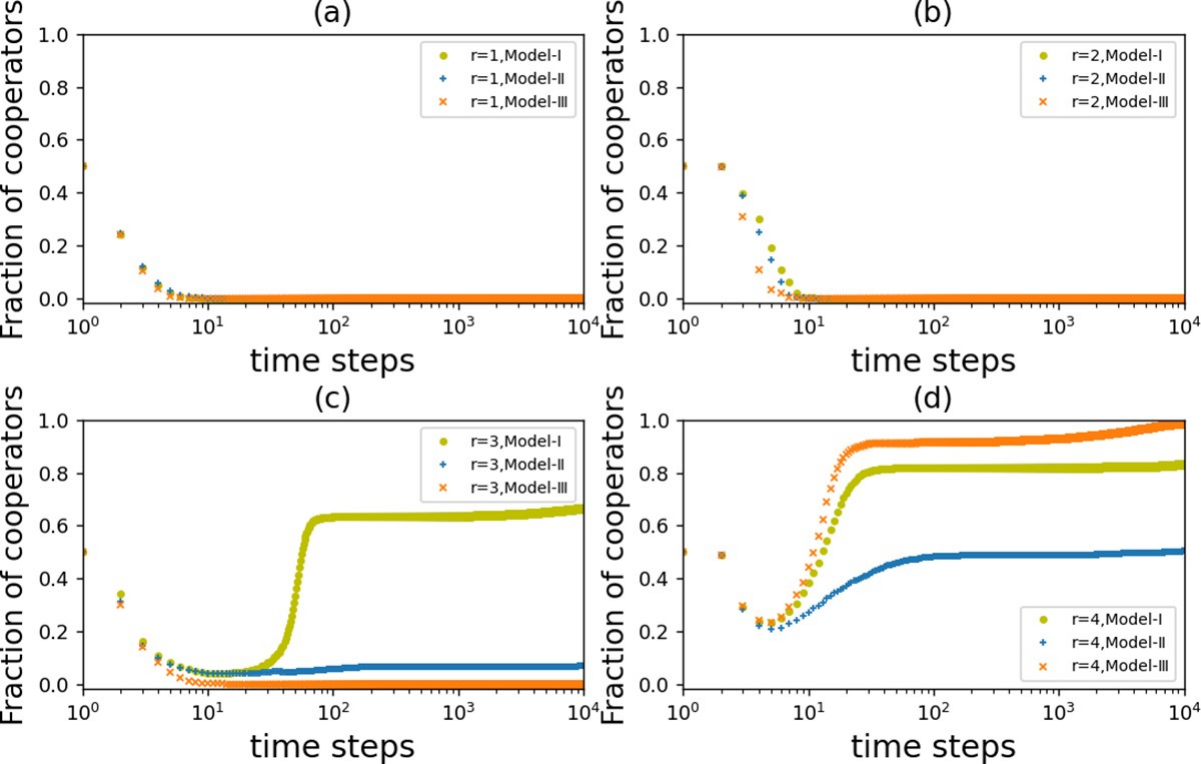
The fraction of cooperators with respect to time for three different partner switching mechanisms. (a) Synergy factor *r =* 1; (b) *r =* 2; (c) *r* = 3; (d) *r* = 4. When *r* = 1 and *r* = 2, the cooperation reward is too low, defectors quickly spread and finally become dominant over the whole population. For *Model-I*, the level of cooperation was higher both when defection dominated (*r = 3*) and cooperation dominated (*r* = 4). *Model-II* and *Model-III* have disadvantages of not being conducive to spreading cooperative and protecting cooperator, respectively. Parameters: *N* = 10000, *T* = 20, *α* = 5, $\hat{k}=4$, *β* = 5, and *ω* = 0.1.

Unlike *Model-I*, *Model-Ⅱ* and *Model-Ⅲ* do not use the average trust information of the known region. Based on the same parameters, we compare the time evolution results of three models at different values of *r*. According to Fig 6(A) and Fig 6(B), when *r* = 1 and *r* = 2, no cooperators survive in all three models. This is because the small synergy factor *r* leads to too low of a payoff from cooperative behavior, so the three forms of topological punishment are not enough to protect the cooperator. When *r* increases to *r* = 3, the cooperators in *Model-I* and *Model-Ⅱ* survive in the network, but all nodes in *Model-Ⅲ* still use the defection strategy, as shown in Fig 6(C). This means that compared with *Model-I* and *Model-Ⅱ*, the random partner switching in *Model-Ⅲ* cannot protect the cooperators well when *r* is small. It is worth noting that although cooperators survived in the evolution results of *Model-I* and *Model-Ⅱ*, the number of cooperators was significantly different between Model-I and *Model-Ⅱ* after the evolution stabilized. The proportion of cooperators in *Model-Ⅱ* dropped rapidly and remained stable at a low value with time, when *r* = 3. However, the proportion of cooperators in *Model-I* increases significantly and remains stable at a high level after a short decline. Therefore, trust-based partner switching among partitioned regions can effectively spread the cooperation strategy while protecting the survival of the cooperators, compared with intermediate region preference. This suggests that even if we limit the amount of information a player can get from the network, taking advantage of the average trust in a known region can still lead to a more suitable partner in the evolution. Finally, we increase *r* to *r* = 4, which is the threshold at which cooperation in a static network starts to dominate under the same conditions. As shown in Fig 6(D), cooperation can rapidly propagate the cooperation strategy to the whole network in the random partner switching of *Model-Ⅲ*, while the cooperation with intermediate region preference in *Model-Ⅱ* is the worst. Therefore, the partner switching model that we propose has the ability to protect cooperators and propagates cooperative strategy under the premise of limited edges.

**Fig 7:**
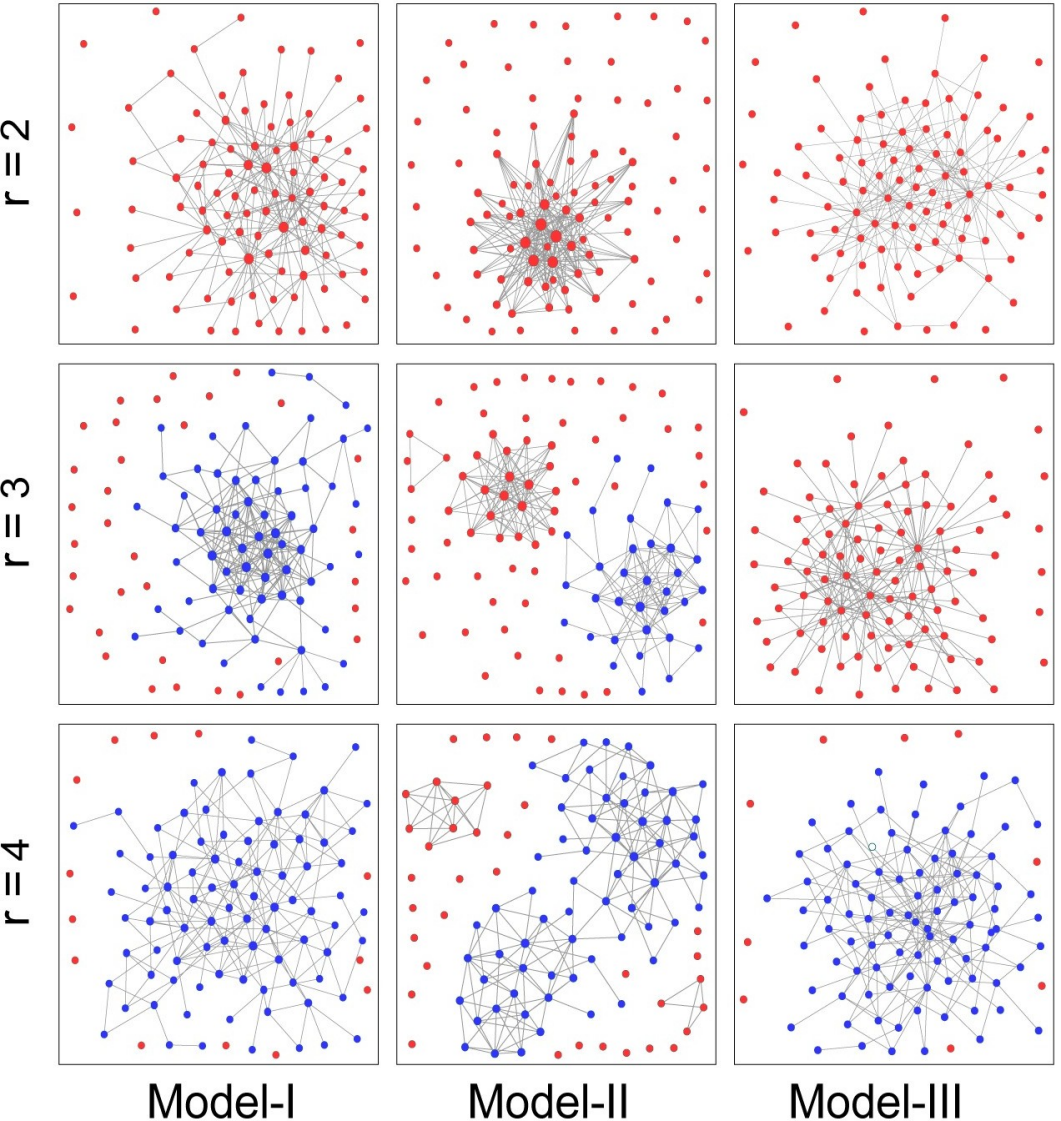
Characteristic snapshots of the network for three partner switching methods with different r, after the cooperation stabilizes. Cooperators and defectors are marked by blue and red, respectively. For *Model-I* and *Model-II*, there are a lot of triangles in the network. For *Model-I* and *Model-III*, networks have strong connectivity. Parameters: *N* = 100, *t* = 10000, *T* = 20, $\hat{k}=4$, *α* = 5, *β* = 5, and *ω* = 0.1.

We further observe how the three models affect the evolution of cooperation by taking a snapshot of the network after the evolution stabilizes. As shown in Fig 7, among the three *r* values, the stable network in *Model-I* maintains good connectivity, and many triangular structures appear in the network. Although a large number of triangular structures also appear in the maximally connected subgraph in *Model-Ⅱ*, there are a large number of isolated defectors in *Model-Ⅱ* and the network connectivity is very poor. *Model-Ⅲ* maintains good connectivity in all three *r* values, but it contains less triangular structures, which means that its network clustering coefficient is not high. Therefore, the differences in the cooperation results of the three models under different *r* can be explained. When *r* = 3, it is not easy to form triangular structures by the random partner switching model in *Model-Ⅲ*, so it does not protect cooperators well. Meanwhile, both the partner switching methods of *Model-I* and *Model-Ⅱ* can form cooperative clusters in the network to resist the invasion of defectors so that cooperators can survive in the evolution. However, the connectivity of *Model-Ⅱ* is poor, and the cooperation strategy is not well spread in the network, which is the reason why the cooperation result of *Model-Ⅱ* is inferior to that of *Model-I*. When cooperation predominates, that is, when *r* = 4, the random partner switching model with best connectivity cooperation results in region-wide cooperation. At the same time, *Model-I* also performs well, taking full advantage of the average trust information of the known region, which allows players to judge the level of cooperation in the environment. From this, we can see that trust-based partner switching among partitioned regions leads to significant promotion of cooperation, which balances the ability to protect partners with the ability to spread cooperative behavior.

### 3.3 Influence of the parameters in trust score

**Fig 8:**
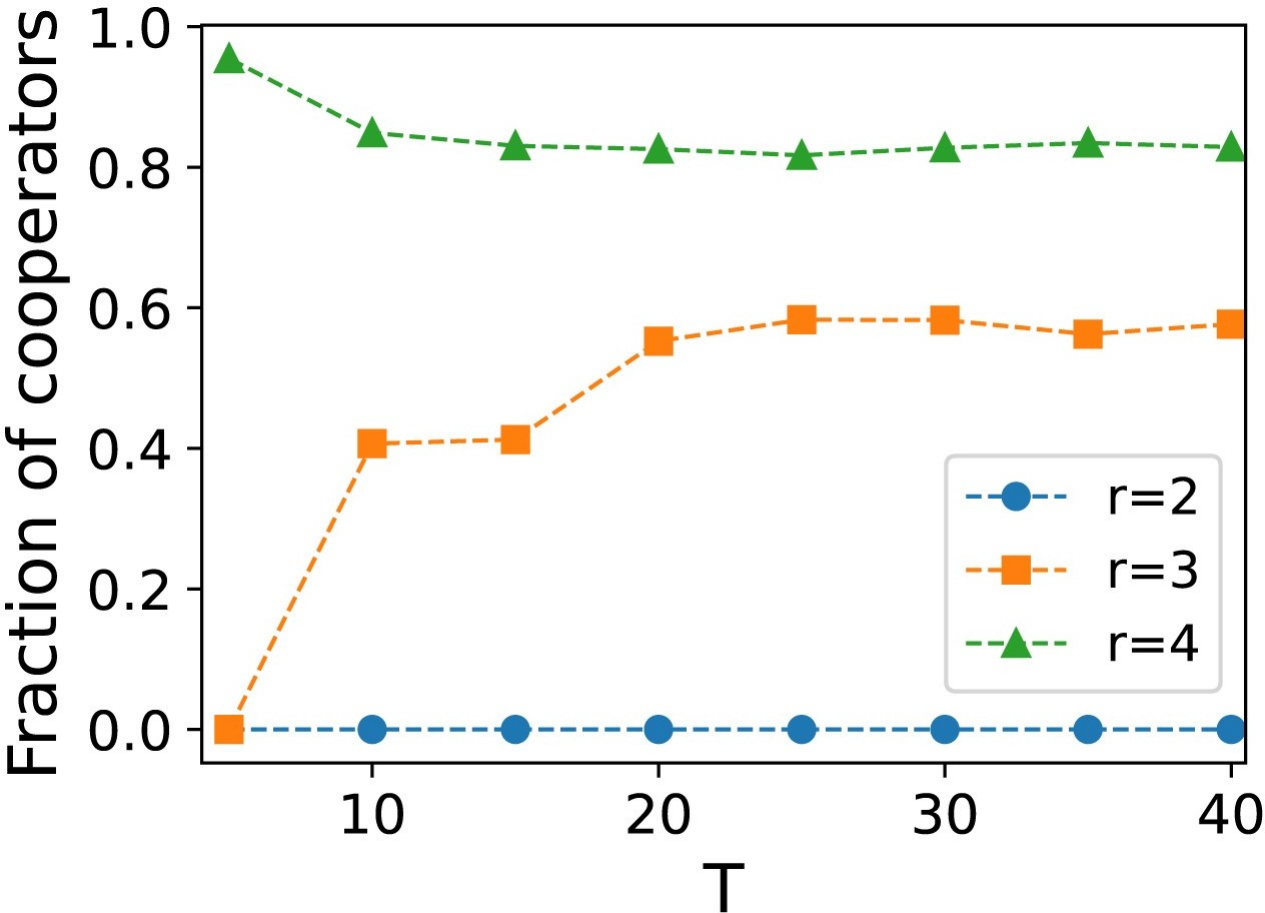
Sensitivity analysis of the trust score threshold *T*. When the trust score threshold *T*≥20, the results of cooperation evolution show a relatively stable trend. *N* = 10000, *t* = 10000, *α* = 5, *β* = 5, and *ω* = 0.1.

**Fig 9:**
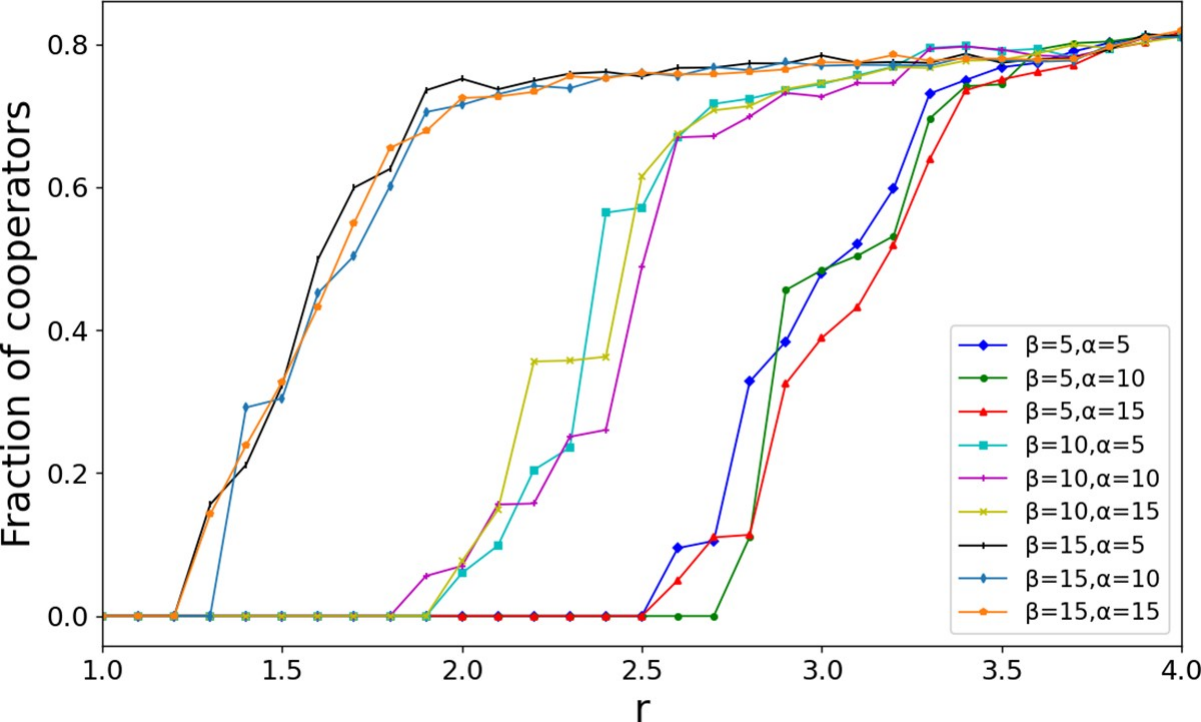
The fraction of cooperators as a function of the synergy factor *r* for different combinations of *α* and *β* with *Model-I* partner switching. The increase of *β* obviously promotes the emergence of cooperation. The change of α has little influence on the cooperation result. Parameters: *N* = 10000, *t* = 10000, *T* = 20, $\hat{k}=4$, and *ω* = 0.1.

We investigate the influence of parameters in trust scores on cooperation results. *T* is the threshold value of a player’s trust score in their neighbors. We investigate whether the results of cooperative evolution are sensitive to the value of threshold. As shown in Fig 8, when *T*≥20, the results of cooperation evolution tend to be stable and do not show the sensitivity to the threshold. This is because when a player has a trust score of -20 for a neighbor, the probability *p* of the player disconnecting from that neighbor, as calculated by Eq 6, is already greater than 0.99. If the *T* value is less than the mean parameter in Eq 6, the probability of the neighbor disconnecting from the dissatisfied neighbor is no more than 0.5. Parameter α and parameter *β* are the two change steps of the trust score, which correspond to the positive and negative effect of the player’s unilateral payoff to the neighbor in a separate game. The increase of *α* will decrease the possibility of edge breaking, whereas the increase of *β* will increase the probability of partner switching. We selected nine different combinations of α and *β* values to observe the effects of the two parameters on the cooperation results. As shown in Fig 9, the increase of *β* can significantly promote cooperation, whereas the effect of *α* on cooperation results is not obvious. Therefore, in this model, the impression of a positive gain from a cooperative act does not have much effect on the cooperative result, but the threat of a loss from defection can effectively promote the cooperation.

**Fig 10:**
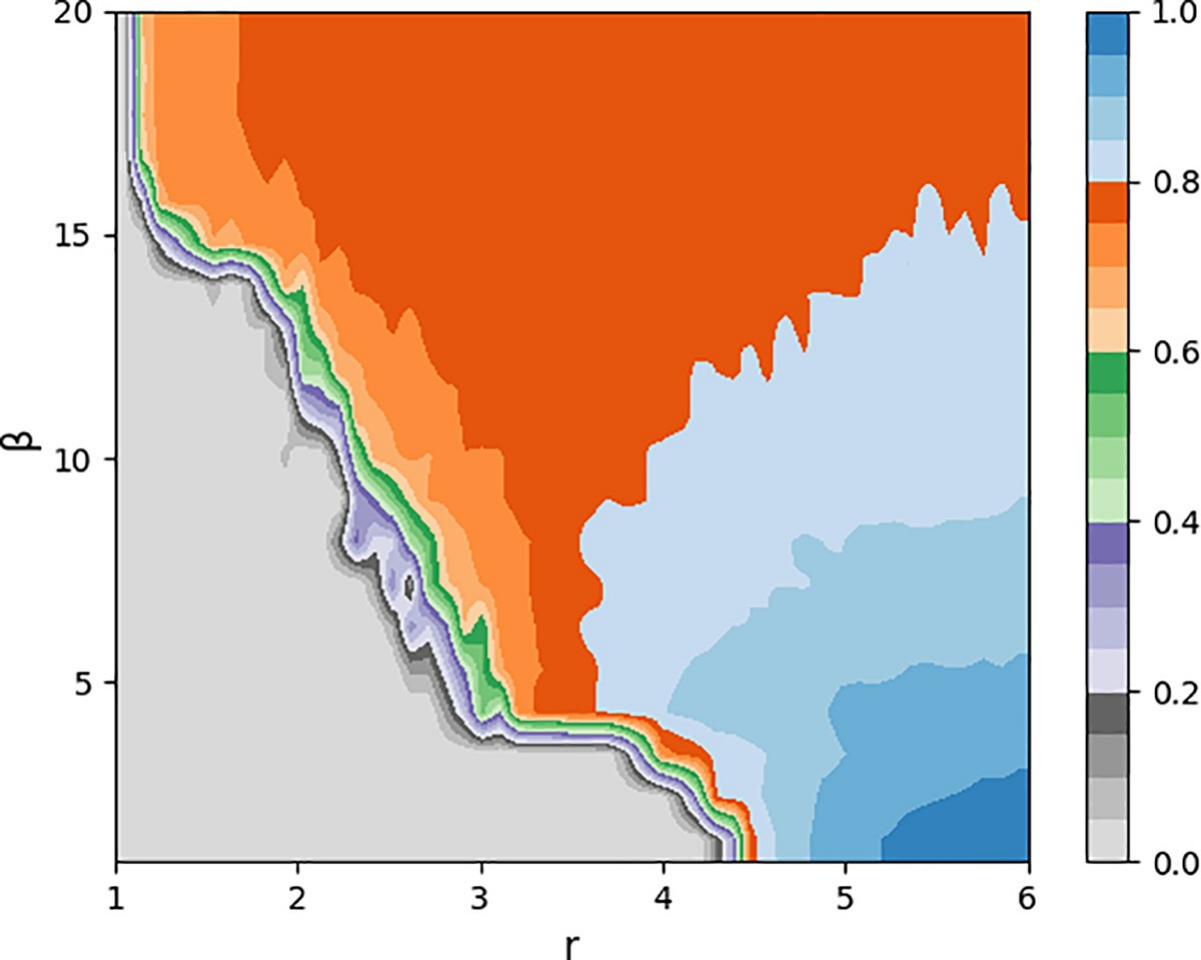
The effects of parameter *β* in partner switching *Model-I*, which is the average fraction of cooperators as a function of (*β*, *r*). When defection dominates (*r*<4), *β* increasing can promote the emergence of cooperation. When cooperation dominates (*r*>4), the increase of *β* will reduce the level of cooperation in the network. Parameters: *N* = 10000, *t* = 10000, *T* = 20, $\hat{k}=4$, *α* = 5 and *ω* = 0.1.

We further investigate the effect of different combinations of *β* and *r* on the cooperation results. As shown in Fig 10, as the parameter *β* increases, the cooperator can survive with a smaller *r*. As *β* increases, more trust scores drop due to defection, which also means that the frequency of partner switching increases. Timely disconnection of a relationship from an unsatisfactory neighbor can improve the advantage of the cooperator to some extent, which has also been proved in many previous studies. However, it is observed from Fig 10 that the increase of *β* for different *r* did not always promote cooperation. When r is large (*r*>4), the increase of *β* will decrease the proportion of cooperators in the whole network. According to Fig 4(A), when *r*>4 is observed, the collaborator can gradually dominate the whole network without the promotion mechanism. In Fig 10, the decrease of global cooperation proportion with the increase of *β* appears at *r* = 4, which is close to the threshold of static network cooperation emergence in Fig 4(A). Therefore, the increase of *β* can preserve partnerships when *r* is low, but it is not conducive to the global diffusion of cooperative strategy when the synergy factor is high. This is because with the increase of partner switching frequency, this model reduces the network connectivity to some extent, so that the cooperation strategy cannot be propagated well.

## 4 Conclusion and discussion

To understand a complex system that is constantly evolving, its structure and dynamics are often studied independently. In recent years, the coevolution of player strategy and network structure has been studied as an important mechanism that promotes cooperation. Many forms and driving factors of coevolution have been studied extensively [26,49,50,52], including reputation [26,52]. Reputation is a non-revenue reward for players who choose to cooperate during evolution, and becomes a social attribute. In the reputation-driven coevolution network, the cooperators attract more neighbors to form indirect reciprocity, which evolves into stable cooperative results. Some studies restrict the spread of player reputation information within a certain neighborhood order, but the historical value of reputation is still obtained by new partners [54]. Reputation as a system reward is defined similarly to social credit policy. However, in some cases, the social reputation of the new partner is not available to the player, and information about the new partners can only be obtained from future games played with them. Therefore, in our model, trust is based on the fact that two players have a game relationship.

Here, we have investigated the coevolution of trust-based strategy updating and partner switching by means of numerical simulations. In the preliminary simulation test, compared with the static network under the same initial conditions, the coevolution network driven by trust score causes cooperation to emerge earlier. If a player gives a neighbor a high trust score, it means that they are satisfied with the payoff brought by the neighbor. Meanwhile, the player disconnects neighbors with a low trust score according to the probability in Eq 6, which is a topological punishment for unsatisfactory neighbors. Two players in a game relationship score each other and build trust. Considering that a player’s trust may vary in magnitude in response to unilateral payoff gains or losses, we introduce a two-step process to describe the change of trust scores in separate games. The simulation results show that for neighbors with a positive unilateral payoff, the change of trust step size does not promote or inhibit the emergence of cooperation. This is because only topological changes are involved in our model, and increased goodwill towards partners does not lead to substantial investment rewards. However, increasing vigilance against defectors can lead to frequent partner switches, which can result in substantial punishment for defectors. Therefore, when the cooperation reward is low (*r<k*), increasing vigilance towards free-riding [12] neighbors can effectively promote the emergence of cooperation in the network. However, it is worth noting that when cooperation predominates (*r>k*), toleration of defectors is more likely to spread cooperation.

When mutually satisfied players give each other positive trust scores, their relationship will be stable and mutually beneficial. Under the conditions of limited edges, how to find trustworthy partners in the network is a problem that needs to be considered. We investigate the influence of partner switching preferences on cooperation results during evolution. Assume that players have no information about the global level of cooperation in the network. Player *x* divides other players in the network into three regions according to the amount of information *x* has mastered. The directly connected nodes are the known region, the indirectly connected second-order neighbors are the intermediate region, and the others are the unknown region. Obviously, the player *x* has mastered the average trust scores for his/her known region, which reflects the average level of cooperation in his/her local environment. The simulation results show that intermediate region preference for partner switching will form a stable trust cluster in the network, thus protecting the partners from a defector’s invasion. A large number of triangular structures appear in trust clusters, and stable game relationships are established between cooperators. However, this strategy weakens the connectivity of the network and is not conducive to the spread of cooperation. In contrast, random partner switching can maintain network connectivity, but it does not protect cooperation well when the synergy factor is low (*r<4*). Meanwhile, trust-based partner switching performs well in both the protection of cooperators and the spread of cooperative strategies. In summary, the trust information in the known region can be used in a reasonable way to find appropriate partners with whom to establish trust and a game relationship. Our primary results shed some light on understanding partner switching among partitioned regions, which is very common in economic regions and economic globalization.

## References

[1] Capraro V, Perc M. Grand challenges in social physics: in pursuit of moral behavior. Frontiers in Physics. 2018;6:107.

[2] Hardin G. 1968 “The tragedy of the commons”, Science 162: 1243–1248. 1968. 5699198

[3] Pennisi E. On the origin of cooperation. American Association for the Advancement of Science; 2009. doi: 10.1126/science.325_1196 19729633

[4] Poundstone W. Prisoner’s Dilemma Doubleday. NY NY. 1992.

[5] Nowak M, Sigmund K. A strategy of win-stay, lose-shift that outperforms tit-for-tat in the Prisoner’s Dilemma game. Nature. 1993;364(6432):56–8. doi: 10.1038/364056a0 8316296

[6] Szabó G, Tőke C. Evolutionary prisoner’s dilemma game on a square lattice. Physical Review E. 1998;58(1):69.

[7] Nowak M. Five Rules for the Evolution of Cooperation. Science. 2006;314(5805):1560–3. doi: 10.1126/science.1133755 17158317 PMC3279745

[8] Groves T, Ledyard J. Optimal Allocation of Public Goods: A Solution to the \"Free Rider\" Problem. Econometrica. 1977;45(4):783–809.

[9] Hauert Christoph, Monte Silvia, De Hofbauer, et al. Volunteering as Red Queen Mechanism for Cooperation in Public Goods Games. Science. 2002. doi: 10.1126/science.1070582 12004134

[10] Rand DG, Nowak MA. The evolution of antisocial punishment in optional public goods games. Nature Communications. 2011;2:434. doi: 10.1038/ncomms1442 21847108 PMC3279747

[11] Santos FC, Santos MD, Pacheco JM. Social diversity promotes the emergence of cooperation in public goods games. Nature. 2008;454(7201):p.213–6. doi: 10.1038/nature06940 18615084

[12] Andreoni J. Why free ride?: Strategies and learning in public goods experiments. Journal of public Economics. 1988;37(3):291–304.

[13] Chen X, Sasaki T, Brännström Å, Dieckmann U. First carrot, then stick: how the adaptive hybridization of incentives promotes cooperation. Journal of the royal society interface. 2015;12(102):20140935. doi: 10.1098/rsif.2014.0935 25551138 PMC4277083

[14] Chen X, Szolnoki A, Perc M. Probabilistic sharing solves the problem of costly punishment. New Journal of Physics. 2014;16(8):083016.

[15] Liu L, Chen X, Szolnoki A. Evolutionary dynamics of cooperation in a population with probabilistic corrupt enforcers and violators. Mathematical Models and Methods in Applied Sciences. 2019;29(11):2127–49.

[16] Liu L, Chen X, Perc M. Evolutionary dynamics of cooperation in the public goods game with pool exclusion strategies. Nonlinear Dynamics. 2019;97(1):749–66.

[17] Liu L, Wang S, Chen X, Perc M. Evolutionary dynamics in the public goods games with switching between punishment and exclusion. Chaos: An Interdisciplinary Journal of Nonlinear Science. 2018;28(10):103105. doi: 10.1063/1.5051422 30384651

[18] Hamilton WD. The genetical evolution of social behaviour. II. Journal of Theoretical Biology. 1964;7(1):17–52. doi: 10.1016/0022-5193(64)90039-6 5875340

[19] Traulsen A, Nowak MA. Evolution of cooperation by multilevel selection. Proceedings of the National Academy of Sciences. 2006;103(29):10952–5. doi: 10.1073/pnas.0602530103 16829575 PMC1544155

[20] Wilson S D. The Group Selection Controversy: History and Current Status. Annual Review of Ecology & Systematics. 1983;14(1):159–87.

[21] Wilson DS. A theory of group selection. Proceedings of the National Academy of Sciences. 1975. doi: 10.1073/pnas.72.1.143 1054490 PMC432258

[22] Axelrod R, Hamilton WD. Evolution of Cooperation: Stanford University Press; 1981.

[23] Nowak Martin, Sigmund Karl. A strategy of win-stay, lose-shift that outperforms tit-for-tat in the Prisoner’s Dilemma game. Nature. 1993. doi: 10.1038/364056a0 8316296

[24] Nowak MA, Sigmund K. Tit for tat in heterogeneous populations. Nature. 1992;355(6357):250–3.

[25] Trivers R. The Evolution of Reciprocal Cooperation. Q Rev Biol. 1971;46:35–57.

[26] Nowak MA, Sigmund K. Evolution of indirect reciprocity by image scoring. Nature. 1998;393(6685):573–7. doi: 10.1038/31225 9634232

[27] Nowak MA, Sigmund K. Evolution of indirect reciprocity. Nature. 2005;437(7063):1291–8. doi: 10.1038/nature04131 16251955

[28] Ohtsuki H, Iwasa Y. The leading eight: social norms that can maintain cooperation by indirect reciprocity. Journal of theoretical biology. 2006;239(4):435–44. doi: 10.1016/j.jtbi.2005.08.008 16174521

[29] Nowak MA, May RM. Evolutionary games and spatial chaos. Nature. 1992;359(6398):826–9.

[30] Ohtsuki H, Hauert C, Lieberman E, Nowak MA. A simple rule for the evolution of cooperation on graphs and social networks. Nature. 2006;441(7092):502–5. doi: 10.1038/nature04605 16724065 PMC2430087

[31] Hauert C, Doebeli M. Spatial structure often inhibits the evolution of cooperation in the snowdrift game. Nature. 2004;428(6983):643–6. doi: 10.1038/nature02360 15074318

[32] Barabási A-L, Albert R. Emergence of scaling in random networks. science. 1999;286(5439):509–12. doi: 10.1126/science.286.5439.509 10521342

[33] Liu Q, Xu Z, Zhang L. Evolutionary public goods game on evolving random networks. Journal of the Korean Physical Society. 2018;72(4):480–4.

[34] Chen X, Wang L. Promotion of cooperation induced by appropriate payoff aspirations in a small-world networked game. Physical Review E. 2008;77(1):017103. 10.1103/PhysRevE.77.017103 18351965

[35] Santos FC, Rodrigues JF, Pacheco JM. Epidemic spreading and cooperation dynamics on homogeneous small-world networks. Physical Review E. 2005;72(5):056128. doi: 10.1103/PhysRevE.72.056128 16383709

[36] Watts DJ, Strogatz SH. Collective dynamics of ‘small-world’networks. nature. 1998;393(6684):440–2. doi: 10.1038/30918 9623998

[37] Assenza S, Gómez-Gardeñes J, Latora V. Enhancement of cooperation in highly clustered scale-free networks. Physical Review E. 2008;78(1):017101. doi: 10.1103/PhysRevE.78.017101 18764081

[38] Ichinose G, Sayama H. Invasion of cooperation in scale-free networks: accumulated versus average payoffs. Artificial Life. 2017;23(1):25–33. doi: 10.1162/ARTL_a_00220 28140631

[39] Lv S, Li J, Mi J, Zhao C. The roles of heterogeneous investment mechanism in the public goods game on scale-free networks. Physics Letters A. 2020;384(17):126343.

[40] Pena J, Volken H, Pestelacci E, Tomassini M. Conformity hinders the evolution of cooperation on scale-free networks. Physical Review E. 2009;80(1):016110. doi: 10.1103/PhysRevE.80.016110 19658777

[41] Perc M. Evolution of cooperation on scale-free networks subject to error and attack. New Journal of Physics. 2009;11(3):033027.

[42] Santos FC, Pacheco JM. Scale-free networks provide a unifying framework for the emergence of cooperation. Physical review letters. 2005;95(9):098104. doi: 10.1103/PhysRevLett.95.098104 16197256

[43] Szolnoki A, Perc M, Danku Z. Towards effective payoffs in the prisoner’s dilemma game on scale-free networks. Physica A: Statistical Mechanics and its Applications. 2008;387(8–9):2075–82.

[44] Pacheco JM, Traulsen A, Nowak MA. Active linking in evolutionary games. Journal of theoretical biology. 2006;243(3):437–43. doi: 10.1016/j.jtbi.2006.06.027 16901509 PMC3279753

[45] Pacheco JM, Traulsen A, Nowak MA. Coevolution of strategy and structure in complex networks with dynamical linking. Physical review letters. 2006;97(25):258103. doi: 10.1103/PhysRevLett.97.258103 17280398 PMC2430061

[46] Santos FC, Pacheco JM, Lenaerts T. Cooperation prevails when individuals adjust their social ties. PLoS Comput Biol. 2006;2(10):e140. doi: 10.1371/journal.pcbi.0020140 17054392 PMC1617133

[47] Zimmermann MG, Eguíluz VM. Cooperation, social networks, and the emergence of leadership in a prisoner’s dilemma with adaptive local interactions. Physical Review E. 2005;72(5):056118. 10.1103/PhysRevE.72.056118 16383699

[48] Zimmermann MG, Eguíluz VM, San Miguel M. Coevolution of dynamical states and interactions in dynamic networks. Physical Review E. 2004;69(6):065102. doi: 10.1103/PhysRevE.69.065102 15244650

[49] Liu C, Shi J, Li T, Liu J. Aspiration driven coevolution resolves social dilemmas in networks. Applied Mathematics and Computation. 2019;342:247–54.

[50] Pichler E, Shapiro AM. Public goods games on adaptive coevolutionary networks. Chaos: An Interdisciplinary Journal of Nonlinear Science. 2017;27(7):073107. 10.1063/1.4991679 28764410

[51] Zhang Y, Liu J, Li A. Effects of Empathy on the Evolutionary Dynamics of Fairness in Group-Structured Systems. Complexity. 2019;2019. doi: 10.1155/2019/4203158 31341377 PMC6656530

[52] Guo H, Chu C, Shen C, Shi L. Reputation-based coevolution of link weights promotes cooperation in spatial prisoner’s dilemma game. Chaos, Solitons & Fractals. 2018;109:265–8.

[53] Sylwester K, Roberts G. Cooperators benefit through reputation-based partner choice in economic games. Biology letters. 2010;6(5):659–62. doi: 10.1098/rsbl.2010.0209 20410026 PMC2936156

[54] Fu F, Hauert C, Nowak MA, Wang L. Reputation-based partner choice promotes cooperation in social networks. Physical Review E. 2008;78(2):026117. doi: 10.1103/PhysRevE.78.026117 18850907 PMC2699261

[55] Sun X, Li Y, Kang H, Shen Y, Peng J, Wang H, et al. Co-Evolution of Complex Network Public Goods Game under the Edges Rules. Entropy. 2020;22(2):199. doi: 10.3390/e22020199 33285973 PMC7516628

